# Investigation of the core binding regions of human Werner syndrome and Fanconi anemia group J helicases on replication protein A

**DOI:** 10.1038/s41598-019-50502-8

**Published:** 2019-09-30

**Authors:** Gyuho Yeom, Jinwoo Kim, Chin-Ju Park

**Affiliations:** Department of Chemistry, Gwangju Institute of Science and Technology, Gwangju, 61005 Republic of Korea

**Keywords:** DNA-binding proteins, DNA damage response, Solution-state NMR

## Abstract

Werner syndrome protein (WRN) and Fanconi anemia group J protein (FANCJ) are human DNA helicases that contribute to genome maintenance. They interact with replication protein A (RPA), and these interactions dramatically enhance the unwinding activities of both helicases. Even though the interplay between these helicases and RPA is particularly important in the chemoresistance pathway of cancer cells, the precise binding regions, interfaces, and properties have not yet been characterized. Here we present systematic NMR analyses and fluorescence polarization anisotropy assays of both helicase-RPA interactions for defining core binding regions and binding affinities. Our results showed that two acidic repeats of human WRN bind to RPA70N and RPA70A. For FANCJ, the acidic-rich sequence in the C-terminal domain is the binding region for RPA70N. Our results suggest that each helicase interaction has unique features, although they both fit an acidic peptide into a basic cleft for RPA binding. Our findings shed light on the protein interactions involved in overcoming the DNA-damaging agents employed in the treatment of cancer and thus potentially provide insight into enhancing the efficacy of cancer therapy.

## Introduction

Werner syndrome protein (WRN) and Fanconi anemia group J protein (FANCJ) are DNA helicases which maintain genomic stability by participating in double-strand break (DSB) repair and interstrand crosslink repair, as well as other DNA processing events^1^. Defects in WRN lead to Werner syndrome, which is characterized by premature aging and high cancer incidence^2^. FANCJ mutations cause Fanconi anemia, early onset breast cancer, and ovarian cancer^3–5^. Both helicases have been considered as anti-cancer targets because of their elevated expression in cancer cells and their ability to overcome DNA-damaging agents in that context^6,7^.

**Figure 1:**
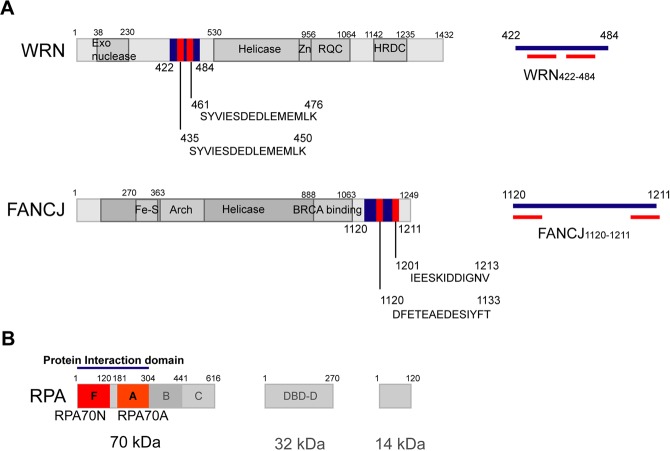
(**A**) Domain structure of WRN and FANCJ. The right panels depict the position of the acidic peptides (WRN_435–450_, WRN_461–476_, FANCJ_1120–1133_, FANCJ_1201–1213_; red) within the polypeptide sequences (WRN_422–484_, FANCJ_1120–1211_; blue) used in this study. (**B**) Three subunits of RPA and its domain structure. Blue bars at the top indicate the general RPA regions known to bind to FANCJ and WRN.

WRN is one of the human RecQ helicases and is composed of an exonuclease, a helicase, a Zn-binding, a RecQ C-terminal (RQC), and a helicase and RNase D C-terminal (HRDC) domain (Fig. 1A). It has 3′-5′ helicase and strand annealing activities along with 3′-5′ exonuclease activity^8,9^. FANCJ is one of a superfamily of 2 iron-sulfur (Fe-S) helicases with 5′-3′ helicase activity (Fig. 1A). Both helicases interact with replication protein A (RPA), and these interactions significantly enhance the DNA unwinding activities of both helicases^10–12^.

RPA is a eukaryotic single-stranded DNA (ssDNA) binding protein, which is composed of subunits RPA70, RPA32, and RPA14. Among the six oligonucleotide binding (OB) folds in RPA, DNA binding domains (DBDs) A, B, and C (in RPA70) and D (in RPA32) mainly bind to ssDNA^13,14^. DBD-F in RPA70’s N terminal domain (RPA70N) and RPA70A are known as regions for protein-protein interaction^15^ (Fig. 1B). In particular, RPA70N interacts with various DNA damage proteins such as ATRIP, RPA9, MRE11, and p53^16,17^. Bloom syndrome protein (BLM), a human RecQ helicase, also interacts with RPA70N^18^. Interestingly, most RPA70N interactors contain acidic-rich sequences and bind to the basic cleft region of RPA70N.

Previous research showed that WRN could unwind longer double-stranded DNA substrates in the presence of RPA, becoming a ‘superhelicase’ when bound to multiple RPAs^12,19^. Studies using truncated constructs showed that a region containing two acidic repeats in human WRN (WRN_424–475_, 52 a.a.) and the N-terminal half of human RPA70 (RPA70N and RPA70A, RPA70_1–308_) were essential for their physical binding^10,12^. These findings piqued our interest because RPA70A is known as the primary ssDNA binding domain of RPA. Also, because mouse WRN contains only one acidic-rich sequence (27 a.a), highly conserved with human WRN, and because it was revealed that 15 a.a. of the acidic-rich region was sufficient to support RPA70N binding^16–18^, we wondered what purpose two acidic repeats might serve in the human WRN-RPA interaction. In order to define the core binding sequence and investigate the binding properties, we tested the binding of various WRN constructs to RPA70N and RPA70A with nuclear magnetic resonance (NMR) spectroscopy and fluorescence polarization anisotropy (FPA) assays.

It has been reported that FANCJ co-localizes with RPA in nuclear foci that contain BRCA1 after DNA damage^11^. It was also revealed that interaction with RPA restores the unwinding activity of FANCJ, which can be blocked by TTAGGG repeat binding factors in the forked duplex telomeric substrate^20^. These results show that the FANCJ-RPA interaction plays an essential role in DNA metabolism, including replication and repair. However, the details of the binding properties, such as the binding region of each partner, have not been studied. Interestingly, FANCJ is the only member of the human 2 Fe-S helicase superfamily that physically interacts with RPA70^11^, and unlike other family members, FANCJ has an unstructured C-terminal region which contains acidic-rich sequences. Two acidic sequences in the C-terminus of FANCJ (residues 1120–1133 and 1201–1213) have similar sequence compositions to known RPA70N interactors such as p53^21^, ATRIP^17^, BLM^18^, SV40^22^, and ETAA1^23^. Based on this, we hypothesized that acidic regions of the C-terminus of FANCJ could interact with RPA70N, and we tested this hypothesis with NMR spectroscopy and FPA assays.

In this study, we performed chemical shift perturbation (CSP) analyses of RPA70N and RPA70A using titrations of various constructs from WRN and FANCJ helicases. We mapped the binding interfaces and measured dissociation constants of each binding pair using FPA assays. We found that the acidic WRN peptide binds not only to RPA70N but also, weakly, to RPA70A. Our FPA analysis showed that the two tandem acidic repeats bind to a dual RPA70N-A construct tighter than the single acidic peptide binds to RPA70N alone. We also found that FANCJ_1120–1133_ specifically interacts with RPA70N, and that two aromatic residues of the sequence are crucial for the binding. Our analysis provides detailed information on the WRN-RPA and FANCJ-RPA interactions that may inform inhibitory strategies for each helicase.

## Results

### Chemical shift perturbation analysis of RPA70N upon binding to WRN_422–484_

**Figure 2:**
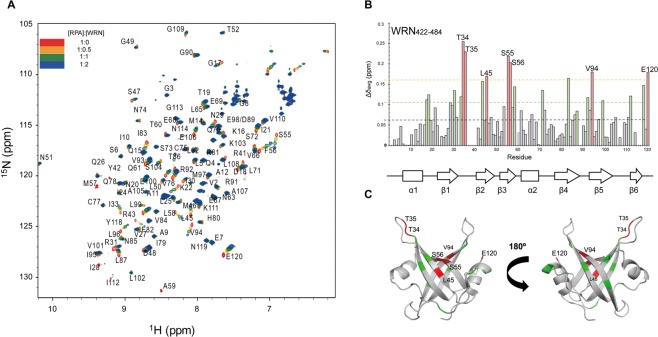
(**A**) Overlaid ^1^H-^15^N HSQC spectra of ^15^N-labeled RPA70N in the absence or presence of increasing molar ratios of WRN_422–484_. (**B**) Averaged chemical shift perturbations (Δδ_avg_) in RPA70N upon interaction with WRN_422–484_. Residues that have Δδ_avg_ greater than one standard deviation (green bars) or greater than two standard deviations (red bars) are indicated. The average CSP (black), one standard deviation over the average (green), and two standard deviations over the average (orange) are shown as dotted lines. (**C**) Residues significantly shifted by WRN_422–484_ are mapped onto the crystal structure of RPA70N (PDB ID: 2B29). Residues perturbed by more than one standard deviation are colored in green and those perturbed by more than two standard deviations are colored in red.

Previous studies have shown that two acidic peptide repeats of WRN located in the N-terminus (WRN_424–475_, 52 a.a.) mainly interact with RPA70_1–326_ ^10,12^. We first performed a series of ^1^H-^15^N HSQC experiments for the CSP analysis of ^15^N-labeled RPA70N with WRN_422–484_ (64 a.a.) to confirm WRN-RPA70N binding. Figure 2A shows the overlaid ^1^H-^15^N HSQC spectra of ^15^N-labeled RPA70N in the absence or presence of increasing molar ratios of WRN_422–484_. Several peaks gradually shifted upon the addition of WRN_422–484_. In the graph of the average CSPs (Δδ_avg_) of RPA70N upon binding to WRN_422–484_, T34, T35, L45, S55, F56, V94, and E120 were perturbed by more than two standard deviations above the average (Fig. 2B). Figure 2C shows those residues in red and residues with Δδ_avg_ greater than one standard deviation above the average in green on the crystal structure of RPA70N (PDB ID: 2B29)^21^. The region is largely overlapped with the basic cleft of RPA70N, which is responsible for the binding of several DNA damage response proteins. Perturbation of the isolated E120 in the flexible C-terminal end is likely due to allosteric effects of peptide binding rather than direct interaction.

### Binding affinity of WRN peptides for RPA70N and RPA70A

**Figure 3:**
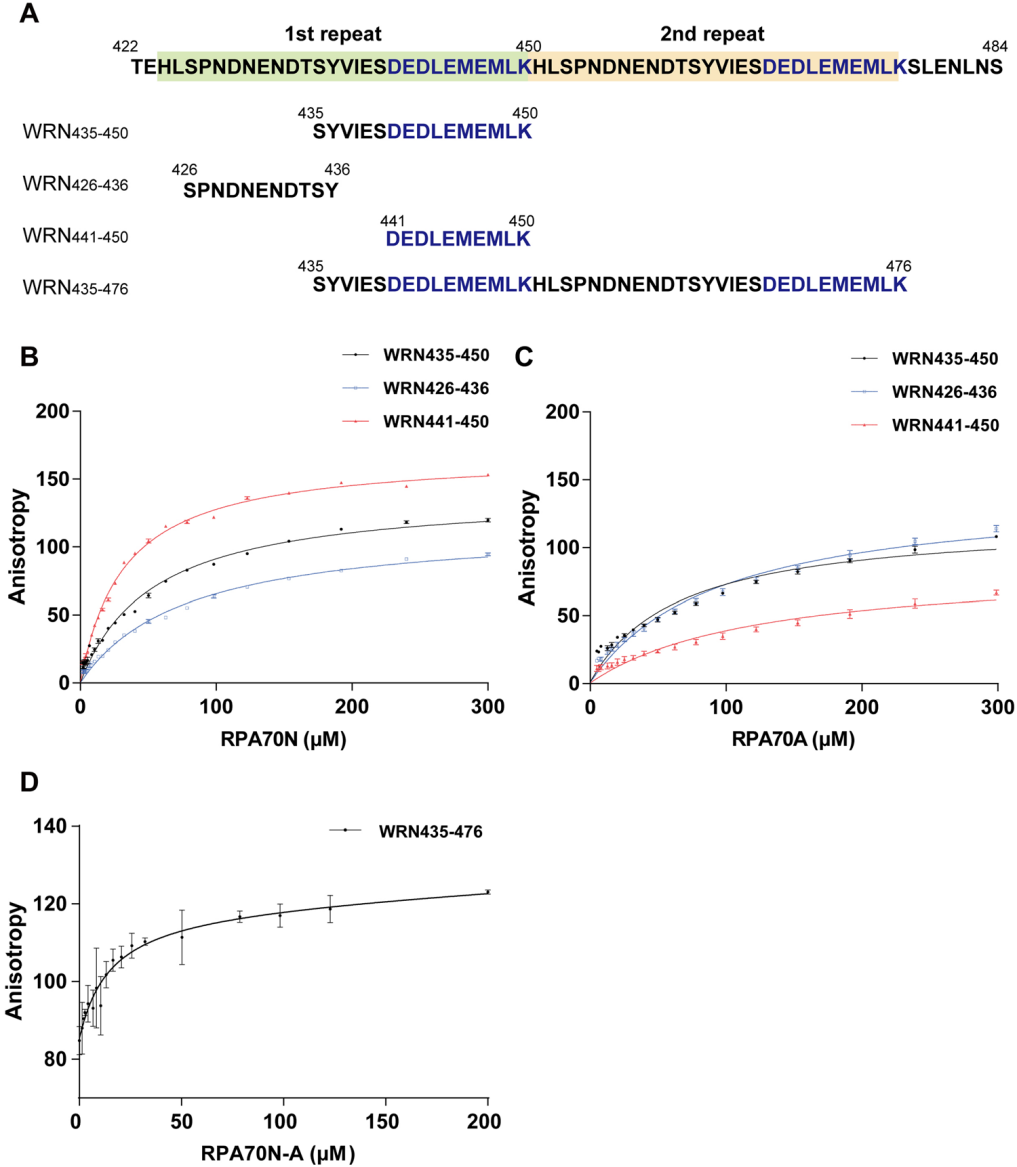
(**A**) Sequences of WRN_422–484_ polypeptide and WRN peptides (WRN_435–450_, WRN_426–436_, WRN_441–450_, and WRN_435–476_) for FPA assays and NMR experiments. (**B**) FPA of WRN peptides upon addition of RPA70N. FPA curves for the WRN peptides are in black (WRN_435–450_), blue (WRN_426–436_), and red (WRN_441–450_). (**C**) FPA of FITC-labeled WRN peptides upon titration with RPA70A. The color scheme is the same as in panel B. (**D**) FPA curve for WRN_435–476_.

Our CSP analysis showed that WRN_422–484_, a region containing two acidic peptide repeats, interacts with RPA70N. In order to define the core region of WRN for RPA binding and examine whether RPA70A also binds to WRN, we performed FPA assays of three FITC-tagged peptides—WRN_435–450_, WRN_426–436_, and WRN_441–450_—upon addition of increasing concentrations of RPA70N or RPA70A (Fig. 3A). We obtained the K_d_ value for each peptide upon titration with RPA70N (Fig. 3B) or RPA70A (Fig. 3C). Table 1 shows the K_d_ values of all the samples we tested. All three peptides bound to RPA70N with K_d_ values in the micromolar range, while they showed weaker interactions with RPA70A than RPA70N. WRN_441–450_, which solely contains acidic residues, showed the lowest K_d_ (29.3 ± 1.2 μM) for RPA70N.

**Table 1:** K_d_ values of FITC-labeled WRN peptides interacting with RPA70N or RPA70A.

	K_d_ (μM)	
	RPA70N	RPA70A
WRN_435–450_	41.4 ± 3.3	65.2 ± 8.1
WRN_426–436_	63.1 ± 5.4	95.8 ± 8.4
WRN_441–450_	29.3 ± 1.2	86.9 ± 17.7
	RPA70N-A	
WRN_435–476_	14.9 ± 5.8	

Interestingly, the lowest K_d_ for RPA70A (65.2 ± 8.1 μM) was observed with WRN_435–450_. This is 1.5-fold weaker than its binding to RPA70N. Our results showed that a single acidic repeat (WRN_435–450_) could bind to both RPA70N and RPA70A with different affinities. This raises the possibility that the first acidic repeat binds to RPA70N and the other repeat binds to RPA70A or *vice versa*. FPA assays of FITC-labeled WRN_435–476_ upon addition of increasing concentrations of RPA70N-(GGGGS)_2_-RPA70A (RPA70N-A) protein was performed to confirm this possibility (Fig. 3D). The K_d_ value of this case was estimated as 14.9 ± 5.8 μM (Table 1). This result showed that the two acidic repeats (WRN_435–476_) could bind to RPA70N-A 2-fold stronger than WRN_441–450_ – RPA70N binding.

### Mapping of the WRN_435–450_ peptide binding surface on RPA70N and RPA70A

Our FPA assays showed that WRN_435–450_ could interact with both RPA70N and RPA70A. In order to map WRN binding surfaces on RPA70N and RPA70A, we performed ^1^H-^15^N HSQC titrations with the WRN_435–450_ peptide. The final pH of the WRN_435–450_:RPA samples at a 2:1 molar ratio was 7.14 because of residual TFA. However, we confirmed that the chemical shifts of the backbone amide protons of RPA70N are almost the same as those at pH 7.4 (Supplementary Fig. S1).

Supplementary Fig. S2 shows the overlaid ^1^H-^15^N HSQC spectra of RPA70N and ^1^H-^15^N cross-peaks of S55, M57, R92, and E120 of RPA70N upon titration with WRN_435–450_. Our results showed S55, M57, N85, R92, and E120 were perturbed by more than two standard deviations above the average (see Supplementary Fig. S2). T34, T35, and L45, which were significantly perturbed by WRN_422–484_, did not have a significant shift change. Even though the absolute magnitudes of Δδ_avg_ were reduced compared to WRN_422–484_ titration, WRN_435–450_ still specifically interacted with the basic cleft region of RPA70N.

In the case of RPA70A, Δδ_avg_ values were small, and the specifically perturbed residues (W212, N214, G219, K220, and E240) were largely overlapped with the ssDNA binding region^14^ (see Supplementary Fig. S2). Also, the binding surfaces were similar to the Rad51-RPA70A interaction^24^.

### Chemical shift perturbation analysis of RPA70N upon binding to FANCJ_1120–1211_

**Figure 4:**
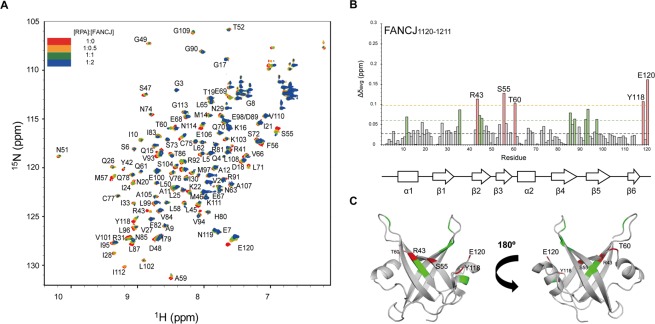
(**A**) Overlaid ^1^H-^15^N HSQC spectra of ^15^N-labeled RPA70N in the absence or presence of increasing molar ratios of FANCJ_1120–1211_. (**B**) Averaged chemical shift perturbations (Δδ_avg_) in RPA70N upon interaction with FANCJ_1120–1211_. (C) Residues significantly shifted by FANCJ_1120–1211_ are mapped onto the crystal structure of RPA70N (PDB ID: 2B29). Interpretation of the color scheme and dashed lines are as described in Fig. 2.

In order to examine our hypothesis that the C-terminal region of FANCJ could specifically engage RPA, we monitored CSPs of the backbone amide peaks of RPA70N in ^1^H-^15^N HSQC spectra upon addition of FANCJ_1120–1211_. As shown in Fig. 4A,B, FANCJ_1120–1211_ mainly perturbed residues in the basic cleft region of RPA70N. R43, S55, T60, Y118, and E120 were significantly changed more than two standard deviations above the average. As in the WRN titration, CSPs of the C-terminal residues (Y118 and E120) are likely due to allosteric changes in the structure. The amplitudes of the Δδ_avg_ values were smaller upon addition of FANCJ_1120–1211_ than of WRN_422–484_, similar to the effects of the BLM peptides^18^. Figure 4C shows the location of FANCJ interacting residues on the structure of RPA70N (PDB ID: 2B29). Once again, they were clustered within the basic cleft of RPA70N.

### Binding affinity of FANCJ peptides for RPA70N and RPA70A

We found that the C-terminal region of FANCJ (FANCJ_1120–1211_) specifically interacts with RPA70N. In order to define the core peptide region for the RPA70 binding, we performed FPA assays of two FITC-labeled peptides, FANCJ_1120–1133_ and FANCJ_1201–1213_, with RPA70N and RPA70A. Supplementary Fig. S3 shows anisotropy changes of FITC-labeled FANCJ peptides with RPA70N. The K_d_ of the RPA70N-FANCJ_1120–1133_ complex was determined to be 40.2 ± 1.8 μM, and the K_d_ of the RPA70N-FANCJ_1201–1213_ complex was estimated as 107.4 ± 4.3 μM (Table 2). This data shows that FANCJ_1120–1133_ has a higher binding affinity for RPA70N than FANCJ_1201–1213_. Both peptides showed weaker binding to RPA70A than to RPA70N (see Supplementary Fig. S3). FANCJ_1120–1133_ and FANCJ_1201–1213_ bind to RPA70A ~4.4-fold and ~2.9-fold weaker than to RPA70N, respectively (Table 2). Our results suggest that the FANCJ-RPA interaction is mainly achieved *via* FANCJ_1120–1133_ and RPA70N.

**Table 2:** K_d_ values of FITC-labeled FANCJ peptides interacting with RPA70N or RPA70A.

	K_d_ (μM)	
	RPA70N	RPA70A
FANCJ_1120–1133_	40.2 ± 1.8	175.8 ± 4.7
FANCJ_1201–1213_	107.4 ± 4.3	318.8 ± 28.9

### Mapping of the FANCJ_1120–1133_ peptide binding surface on RPA70N

**Figure 5:**
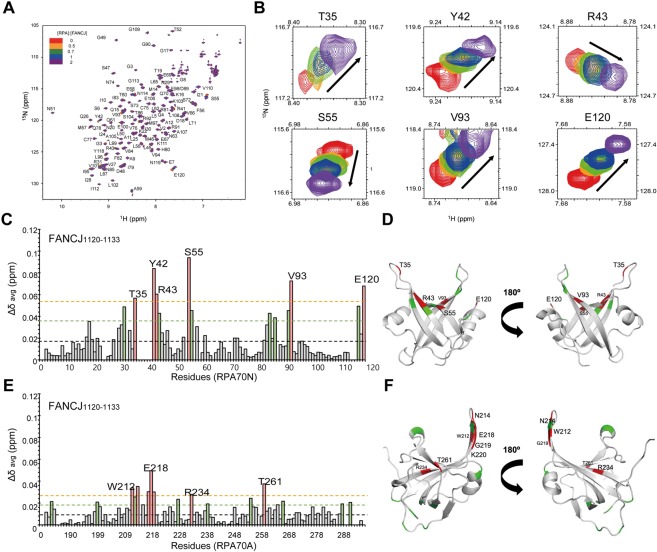
(**A**) Overlaid ^1^H-^15^N HSQC spectra of ^15^N-labeled RPA70N in the absence or presence of increasing molar ratios of FANCJ_1120–1133_. (**B**) ^1^H-^15^N cross-peaks of T35, Y42, R43, S55, V93, and E120 of RPA70N upon titration with FANCJ_1120–1133_. Chemical shift perturbations in (**C**) RPA70N and (**E**) RPA70A upon interaction with FANCJ_1120–1133_. Residues significantly shifted by FANCJ_1120–1133_ are mapped onto the crystal structure of (**D**) RPA70N (PDB ID: 2B29) and (**F**) RPA70A (PDB ID: 1JMC). Interpretation of the color scheme and dashed lines are as described in Fig. 2.

In order to map the FANCJ_1120–1133_ binding surface on RPA70N, we performed a CSP analysis. Figure 5A shows the overlaid ^1^H-^15^N HSQC spectra of RPA70N upon titration with FANCJ_1120–1133_ and Fig. 5B shows ^1^H-^15^N cross-peaks of the most perturbed residues. Figure 5C shows the Δδ_avg_ of RPA70N upon FANCJ_1120–1133_ binding. The magnitudes of Δδ_avg_ were comparable to the values observed by the binding of the longer construct, FANCJ_1120–1211_. This implies that regions other than FANCJ_1120–1133_ do not contribute much to RPA70N binding. T35, Y42, R43, S55, V93, and E120 showed significant perturbations and mostly overlapped with the FANCJ_1120–1211_ binding surface in the basic cleft of RPA70N (Fig. 5D). This data supports that FANCJ_1120–1133_ is the main RPA70N binding region, consistent with our FPA data. Figure 5E shows the Δδ_avg_ of RPA70A upon titration with the same peptide. The small Δδ_avg_ values are consistent with the relatively large K_d_ value (175.8 ± 4.7 μM) determined by our FPA assay. Residues with relatively large changes are in locations similar to as those involved in WRN_435–450_ binding (Fig. 5E,F).

We also performed a titration of FANCJ_1201–1213_ into samples of RPA70N and RPA70A. Neither RPA domain showed significant chemical shift changes (data not shown). This is consistent with the conclusion that FANCJ_1120–1133_ is the main interaction partner of RPA70N. In contrast to the two acidic repeats of WRN, both of which can interact with both RPA70N and RPA70A, FANCJ_1201–1213_, the second acidic region, did not contribute to RPA70 interaction.

### Y1131 and F1132 of FANCJ are critical residues for RPA70N binding

**Figure 6:**
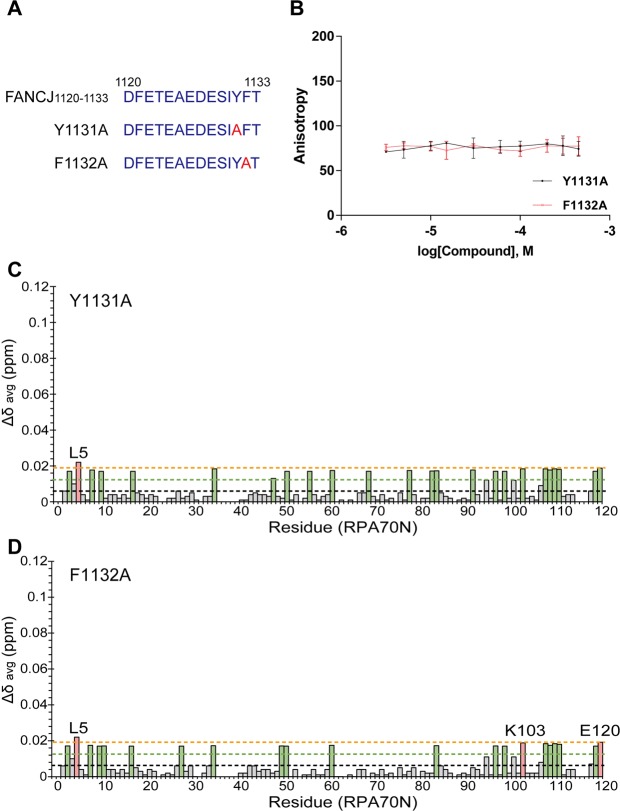
(**A**) Sequences of FANCJ_1120–1133_ and mutated FANCJ peptides (Y1131A and F1132A) for FPA assays and NMR experiments. (**B**) Competitive inhibition of FITC-FANCJ_1120–1133_ binding to RPA70N by mutated FANCJ peptides (Y1131A, circles with a black line; F1132A, squares with a red line). CSPs in RPA70N upon interaction with (**C**) Y1131A and (**D**) F1132A. Interpretation of the color scheme and dashed lines are as described in Fig. 2.

Previous experiments with an ATRIP-based unnatural peptide (DFTADDLEEWFAL) showed that the aromatic residues in the C-terminus of the peptide improved its binding affinity for RPA70N^25^. FANCJ possesses two aromatic residues, Y1131 and F1132, at the end of the first acidic repeat. In order to investigate the effects of these aromatic residues on RPA70N binding by FANCJ, we prepared point mutants, Y1131A and F1132A, of FANCJ_1120–1133_ (Fig. 6A). In an FPA competition assay with FITC-labeled FANCJ_1120–1133_ and RPA70N, the fluorescence signal was not changed with increasing amounts of Y1131A and F1132A (Fig. 6B). This data showed that neither mutant could compete against the wild-type sequence for binding to RPA70N. We also performed ^1^H-^15^N HSQC experiments on ^15^N-labeled RPA70N titrated with both mutants. Figure 6C,D show the Δδ_avg_ of RPA70N upon binding to Y1131A and F1132A, respectively. Strikingly, almost no significant chemical shift changes were observed, not only in the basic cleft region, but across the entire protein. Our FPA competition assay and CSP analysis show that both mutants have much lower affinities for RPA70N compared to the wild-type peptide. This suggests that both aromatic residues, Y1131 and F1132, at the C-terminal end of FANCJ_1120–1133_ are crucial for RPA70N binding.

## Discussion

In this study, we investigated RPA’s interactions with peptides from human helicases, WRN and FANCJ, to identify core sequences for RPA binding. Our CSP analysis showed that WRN_422–484_, which contains two full acidic repeats, specifically interacts with RPA70N *via* the basic cleft region. We also monitored significant CSPs in the basic cleft region of RPA70N upon binding to WRN_435–450_, which contains only one acidic repeat. Even though the binding affinity of WRN_435–450_ for RPA70N is weaker than previously reported cases of BLM peptides^18^, WRN has two tandem RPA binding sites that can compensate for the lower affinity. At the same time, WRN_435–450_ binds to RPA70A with a K_d_ of 65.2 ± 8.1 μM, as determined by FPA assay. This suggests that WRN_435–450_ could interact with both RPA70N and RPA70A, which are connected with a flexible linker. Thus, the proximity of the two binding sites (WRN_435–450_ and WRN_461–476_) could enhance the overall binding affinity compared to that of the individual sequences. The low K_d_ value (14.9 ± 5.8 μM) of WRN_435–476_ with RPA70N-A measured in this study strongly supports this hypothesis. Our data suggest that the WRN-RPA interaction is a multivalent binding, where RPA70N serves as the primary binding site with higher affinity and RPA70A is the secondary binding site. This is consistent with previous research showing several RPA binding partners interacting through multiple contact points, with one contact *via* RPA70N or RPA32C, and a secondary weaker contact within the RPA70AB domain^26^.

Regarding RPA70A’s binding affinity for WRN_435–450_, it is higher than for Rad51, but both of them have much lower affinity compared to the ssDNA-RPA70A complex^26^. This implies that the interaction may not occur with ssDNA-bound RPA. However, more investigations are necessary to reveal the complex interactions between WRN, RPA, and DNA substrates.

Even though the physical and functional interaction between FANCJ and RPA was reported^11^, their binding surfaces were not revealed yet. We hypothesized that the acidic-rich sequence in the unstructured C-terminal region of FANCJ could be a candidate for RPA70N binding based on the unique RPA binding property of FANCJ among superfamily of 2 Fe-S helicases and the sequence conservation^16,18,25^. Our NMR and FPA results clearly showed that one of the candidates, FANCJ_1120–1133_, could specifically interact with RPA70N *via* the basic cleft region. The binding had a dissociation constant of about 40 μM, which is similar to the WRN_435–450_-RPA70N interaction. This result suggests that FANCJ_1120–1133_ binds to RPA70N stronger than RAD9, MRE11, and p53, but weaker than BLM and ATRIP^16^. We also found that two aromatic residues, Y1131 and F1132, in the C-terminus of FANCJ_1120–1133_ are critical for RPA70N binding. This suggests that the FANCJ-RPA interaction is not only electrostatic but also hydrophobically tuned, consistent with the results for FANCJ_1201–1213_, which does not have aromatic residues at the C-terminal end and has a very low affinity for RPA70N.

It has recently been recognized that weak and transient protein-protein interactions, with a K_d_ in the micromolar to millimolar range, are important for the cell’s signaling, regulatory, and stress response mechanisms^27–29^. Accordingly, RPA-mediated protein-protein interactions should not be too strong or persistent, because RPA must interact with the appropriate partner depending on the cellular conditions, such as DNA damage response or repair processes. In this context, the modest binding affinities of RPA-WRN and RPA-FANCJ could be physiologically relevant.

Supplementary Fig. S4 shows the sequence comparison of the RPA70N binding regions of BLM, ETAA1, ATRIP, WRN, and FANCJ. The acidic-rich region in the middle combined with the distribution of hydrophobic residues is known to be crucial for RPA binding. Both FANCJ_1120–1132_ and WRN_437–449_ share these common features. We also compared the binding surfaces on RPA70N for each acidic peptide from WRN, FANCJ, and BLM^18^ (see Supplementary Fig. S4). While residues near S55 participate in the binding of all three proteins, the T60 site does not appear to make contact with the WRN and FANCJ peptides. Thus, the BLM peptides make contact over a relatively wider area. This could be related to the fact that BLM peptides have lower K_d_ values. We also performed docking simulations for WRN_441–450_–RPA70N and FANCJ_1120–1133_-RPA70N on the CABS-Dock Webserver^30^ (see Supplementary Tables S1 and S2). Supplementary Fig. S4 shows the representative model of each complex. Both peptides are located in the basic cleft region between two loops.

While WRN binds to RPA in its N-terminal region, RPA binding region of FANCJ (FANCJ_1120–1133_) is located at the C-terminal region of the protein. A previous report showed that the spatial position of RPA70N is important for optimal stimulation of WRN’s helicase activity from the 3′ to 5′ direction^31^. The relative orientations of the helicases to RPA may depend on the location of the binding regions. We hypothesize that the opposite directionality of FANCJ’s helicase activity compared to BLM and WRN may be related to RPA’s binding in its C-terminus.

In summary, we investigated whether acidic peptides of WRN and FANCJ bind to RPA70N or RPA70A through NMR spectroscopy and FPA assays. Peptides of both proteins bound to RPA70N with K_d_s in the micromolar range, and we identified FANCJ_1120–1133_ as a novel RPA70N binding site. Tandem acidic repeats of WRN mediate multi-domain binding. Our study provides valuable information on RPA’s interactions with WRN and FANCJ helicases, which may be useful for developing therapeutic strategies for cancer treatment.

## Methods

### Sample preparation

We used five fluorescein isothiocyanate (FITC)-labeled peptides (WRN_435–450_, WRN_426–436_, WRN_441–450,_ FANCJ_1120–1133_, and FANCJ_1201–1213_). All were purchased from AnyGen (Gwangju, Korea). These peptides were purified using HPLC with acetonitrile containing 0.05% TFA (trifluoroacetic acid) to 95% purity. Three unlabeled peptides (WRN_435–450_, FANCJ_1120–1133_, and FANCJ_1201–1213_) were used for NMR experiments. These were also purchased from AnyGen and purified with the same method.

RPA70N and RPA70A and a tandem construct comprising RPA70N followed by RPA70A with (GGGGS)_2_ linker were subcloned into a pET15b vector and transfected into BL21 (DE3) cells. The proteins were overexpressed and purified as described previously^16,32^. ^15^N-labeled proteins were obtained by growing cells in M9 media containing ^15^NH_4_Cl and unlabeled D-glucose. FANCJ_1120–1211_, WRN_422–484_, and WRN_435–476_ were subcloned into a pET His6 TEV LIC cloning vector (2B-T) (a gift from Scott Gradia, Addgene plasmid #29666) and then transfected into BL21 (DE3) and Rosetta (DE3) cells, respectively. The cells were grown at 37 °C to an OD_600_ of 0.5–0.6, at which time IPTG was added to a final concentration of 1.0 mM. Cells were incubated for an additional 20 h at 18 °C. His-tagged WRN and FANCJ proteins were purified using a Ni-NTA column (Elpis Biotech, Korea) and eluted with elution buffer (50 mM NaH_2_PO_4_, 300 mM NaCl, 300 mM imidazole). All proteins were loaded onto a Superdex 75-pg FPLC column (GE Healthcare) pre-equilibrated with 20 mM HEPES (pH 7.4), 100 mM NaCl, and 1 mM DTT for further purification.

### NMR experiments

^1^H-^15^N HSQC experiments were performed using a Bruker 900 MHz NMR spectrometer equipped with a cryogenic probe (KBSI, Ochang). We used Bruker basic pulse sequence ‘hsqctf3gpsi2’. The detailed parameters are as follows: Size of Free Induction Decay (TD), 2k (^1^H dimension)/192 (^15^N dimension); Size of real spectrum (SI), 2k/1k. ^15^N-labeled RPA70N and RPA70A were dissolved in 20 mM HEPES (pH 7.4), 100 mM NaCl, and 1 mM DTT at 0.3 mM. For WRN and FANCJ titrations, WRN_422–484_ and FANCJ_1120–1211_ were added at molar ratios of 0, 0.5, 1.0, and 2.0, and WRN_435–450_, FANCJ_1120–1133_, and FANCJ_1201–1213_ were added at molar ratios of 0, 0.1, 0.5, 0.75, 1.0, and 2.0. All experiments were performed at 298 K. Published amide chemical shifts of RPA70N and RPA70A were used to analyze CSP^33,34^. Topspin was used to process the NMR spectra, and data analyses were performed with Sparky^35^. The average chemical shift changes (Δδ_avg_) were calculated according to equation [1],

1 $$\Delta {\delta }_{avg}=\sqrt{{(\Delta {\delta }_{H})}^{2}+{(\Delta {\delta }_{N}/5.88)}^{2}}$$

where Δδ_H_ and Δδ_N_ are the amide proton and nitrogen resonance chemical shift changes, respectively. Residues with changes greater than one or two standard deviations from the average CSP were considered to be significantly perturbed.

### Fluorescence polarization anisotropy experiments

A FITC label with a 6-aminohexanoic acid spacer at the N-terminus of WRN and FANCJ peptides was used for the FPA assays. The fact that FITC does not have a substantial effect on the interaction between RPA70 and the peptides was confirmed by an experiment comparing the affinity of FITC-labeled and unlabeled ATRIP peptide, which is known to bind with RPA70N^16^. Because several acidic peptides for RPA binding have been tested with FITC labeling^16,18,25^, we used the same fluorescent probe for our assays.

Increasing concentrations (0–300 μM) of RPA70N (or RPA70A) in a total 50 μL of assay buffer (20 mM HEPES, 100 mM NaCl, 1 mM DTT, pH 7.4) and 50 nM FITC-labeled peptides were mixed in Corning 96-well black plates (polystyrene, non-treated, flat bottom) and equilibrated for ≥1 h at 25 °C. NHS-Fluorescein (Thermo Fisher, USA) conjugated isothiocyanate (FITC)-labeled protein (WRN_435–476_) and increasing concentrations of RPA70N-A (0–200 μM) were prepared in the same manner. Samples were excited at a wavelength of 485 nm and emission was detected at a wavelength of 528 nm using Cytation5 (BioTek) and Gene5 software (GIST, Gwangju). The emission polarization anisotropy was calculated as described in a previous report^16^. Data analyses were performed using GraphPad Prism version 7.01 (GraphPad Software, La Jolla, CA, USA, www.graphpad.com). At increasing concentrations of RPA70N or RPA70A, anisotropy curves were plotted. All experiments were repeated three times, and each dissociation constant (K_d_) and its standard error was calculated from the ‘one-site specific’ fitting model.

To compare the binding affinity of RPA70N and FANCJ_1120–1133_ with that of the FANCJ mutants (Y1131A and F1132A), an FPA competition assay was performed. To 6 μM RPA70N and 500 nM FITC-FANCJ_1120–1133_ in 200 μM assay buffer, 0–500 μM Y1131A or F1132A were added and equilibrated as described above. The ‘log[Inhibitor] *vs*. response – variable slope (four parameters)’ method of the GraphPad software was applied to the plot. The equation used for the fitting is as follows:

$$Y=Bottom+\frac{Top-Bottom}{1+{10}^{(LogI{C}_{50}-X)\times HillSlope}}$$

Top and Bottom values are plateaus in the units of the Y axis. IC_50_ is the inhibitor concentration that generates a response half way between Bottom and Top. HillSlope represents the steepness of the curve.

However, the fitting was not possible because unlabeled mutant peptides failed to compete with the labeled one (wild-type).

## Supplementary information

Supplementary Figures, Methods, and table
