# Supplementary material for: Investigation of the core binding regions of human Werner syndrome and Fanconi anemia group J helicases on replication protein A
Source: Sci Rep. 2019 Sep 30;9:14016. doi: 10.1038/s41598-019-50502-8 (PMC6768877; doi:10.1038/s41598-019-50502-8)
Supplement: Supplementary file 1 — Supplementary Figures, Methods, and table [file 41598_2019_50502_MOESM1_ESM.docx]

**Investigation of the core binding regions of human Werner syndrome and Fanconi anemia group J helicases on replication protein A**

**Gyuho Yeom^+^, Jinwoo Kim^+^, and Chin-Ju Park^*^**

Department of Chemistry, Gwangju Institute of Science and Technology, Gwangju, 61005, Korea

*correspondence to : [cjpark@gist.ac.kr](mailto:cjpark@gist.ac.kr).

^+^ These authors contributed equally to this work.

**Supplementary Figures**

Figure S1.


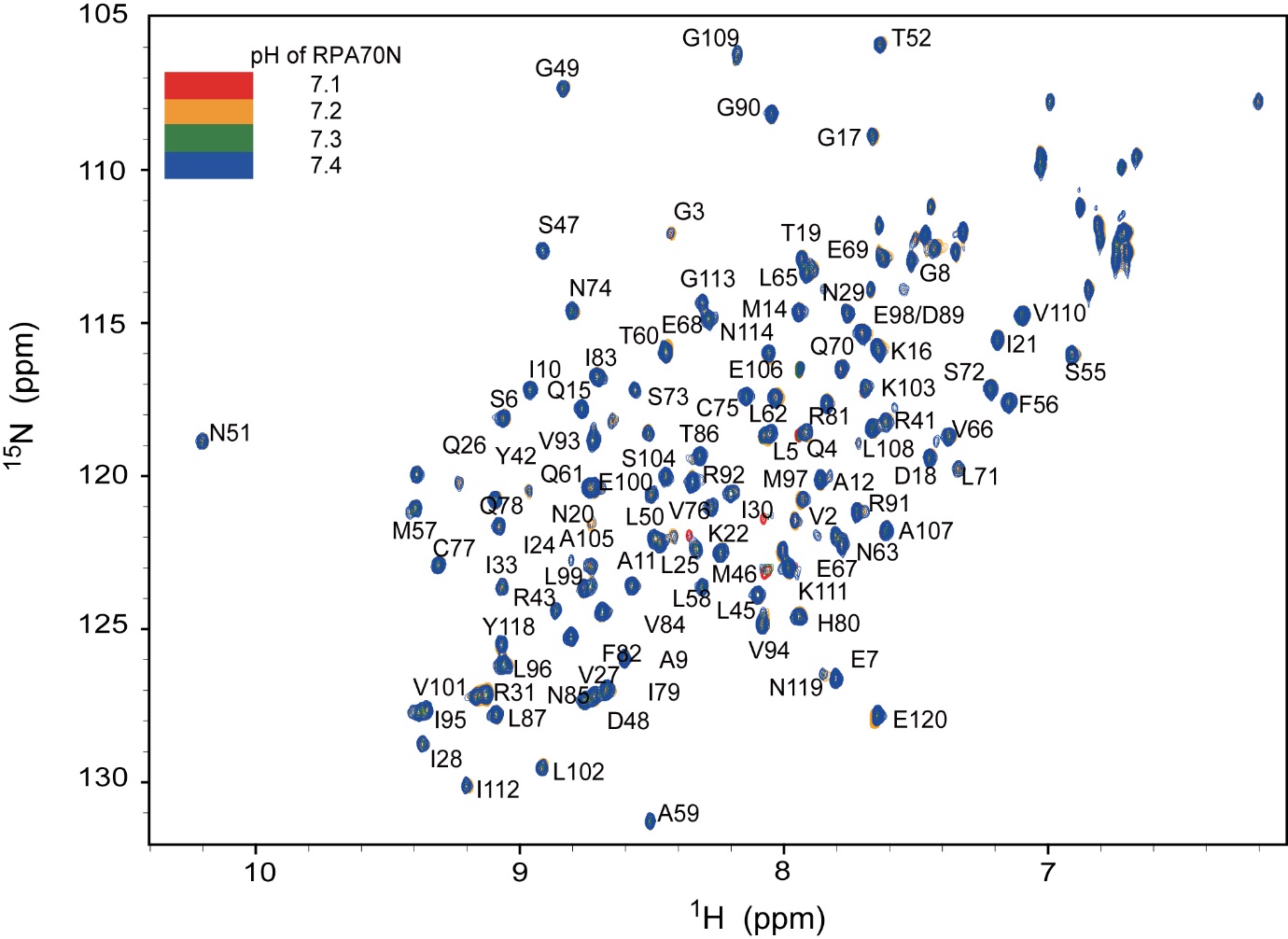
 Figure S1. Overlaid ^1^H-^15^N HSQC spectra of ^15^N-labeled RPA70N at different pHs.

Figure S2.


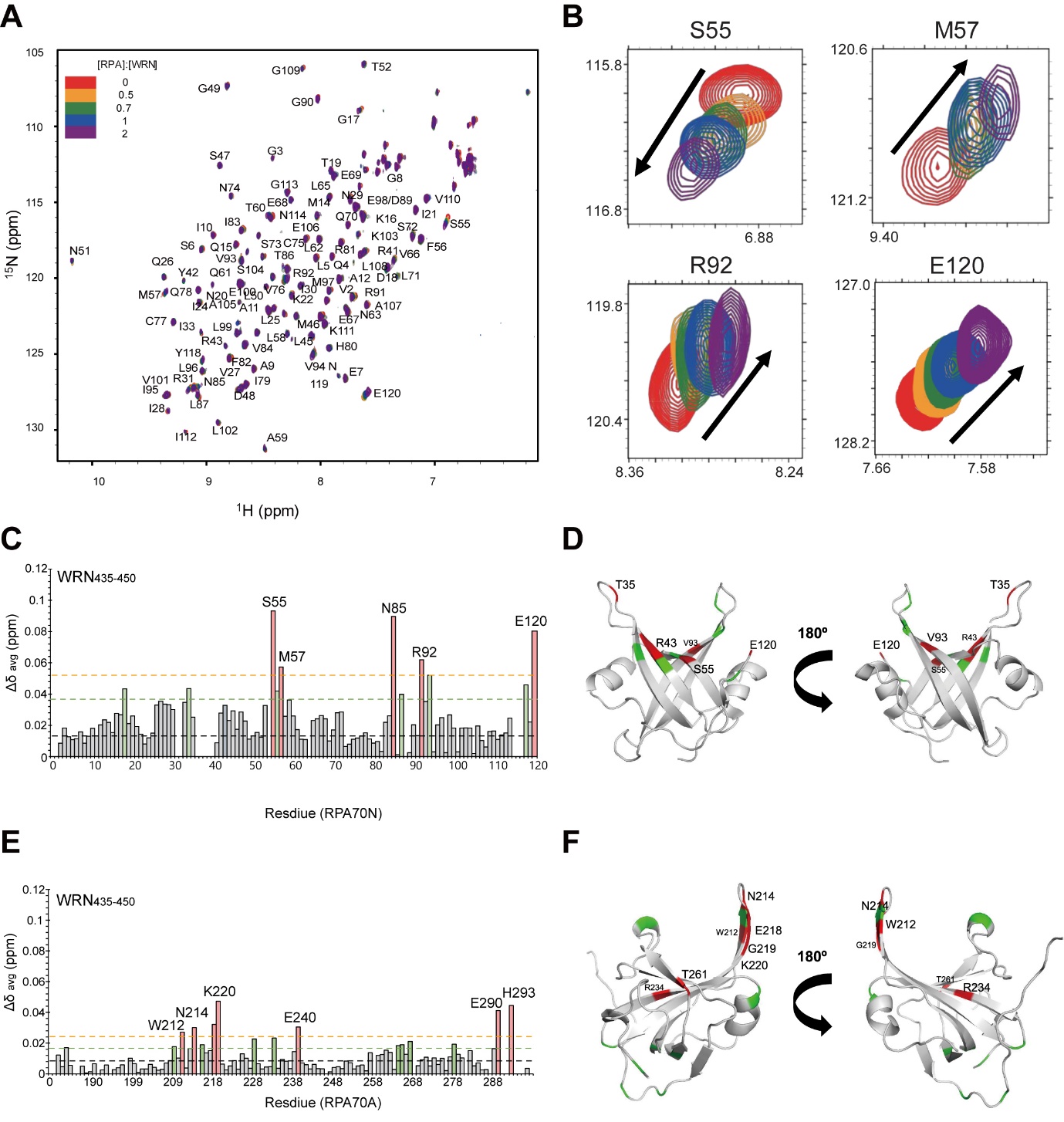
 Figure S2. (A) Overlaid ^1^H-^15^N HSQC spectra of ^15^N-labeled RPA70N in the absence or presence of increasing molar ratios of WRN_435-450_. (B) ^1^H-^15^N cross-peaks of S55, M57, R92, and E120 of RPA70N upon titration with WRN_435-450_. Chemical shift perturbations in (C) RPA70N and (E) RPA70A upon interaction with WRN_435-450_. Residues significantly shifted by WRN_435-450_ are mapped onto the crystal structure of (D) RPA70N (PDB ID: 2B29) and (F) RPA70A (PDB ID: 1JMC). Interpretation of the color scheme and dashed lines are as described in Figure 2.

Figure S3.


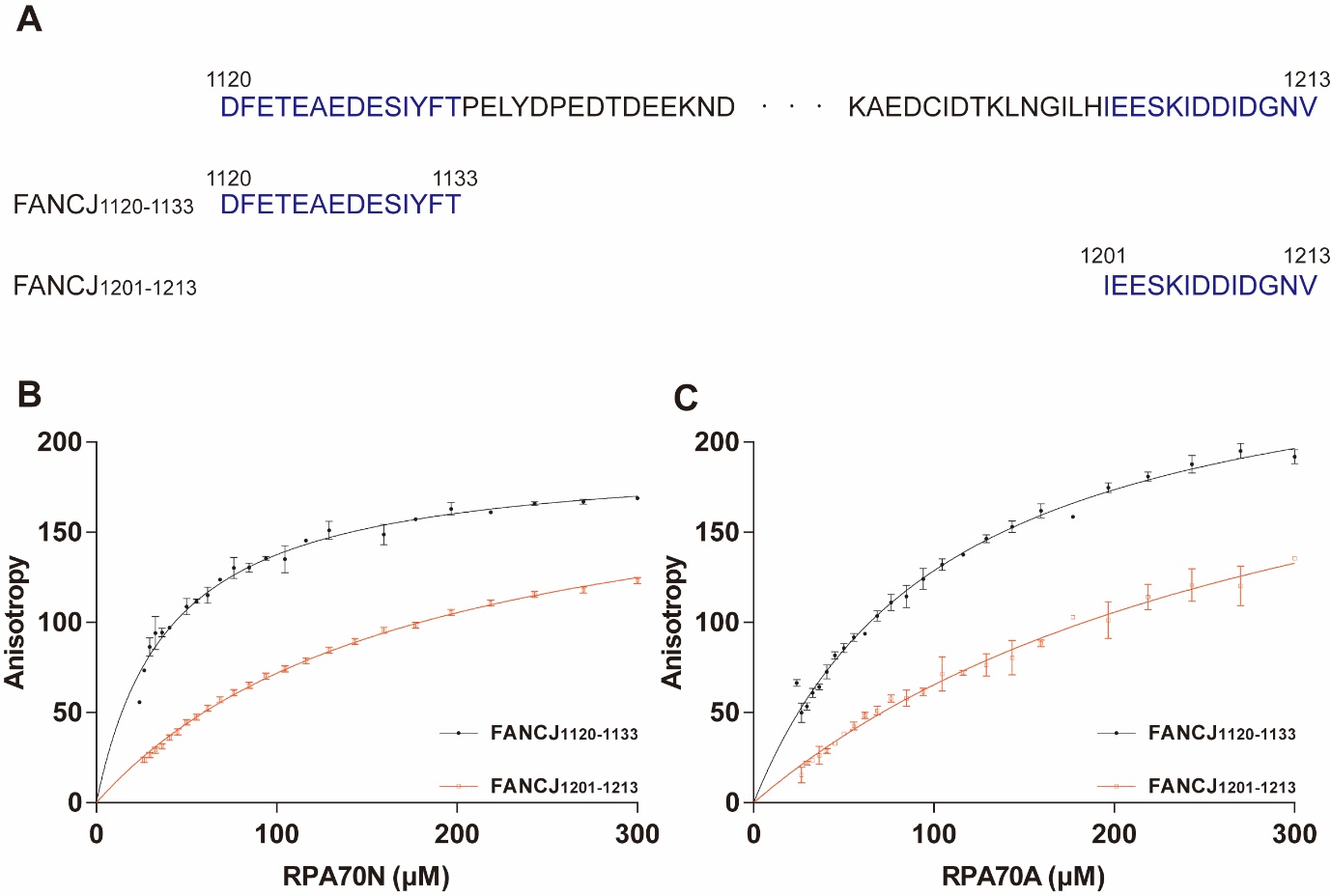


Figure S3. (A) The sequence of the FANCJ_1120-1213_ polypeptide and the two FANCJ acidic peptides (FANCJ_1120-1133_, FANCJ_1201-1213_) used in FPA assays and NMR experiments. FPA of FANCJ peptides upon addition of (B) RPA70N and (C) RPA70A. FPA curves are shown in black (FANCJ_1120-1133_) or red (FANCJ_1201-1213_).

Figure S4.


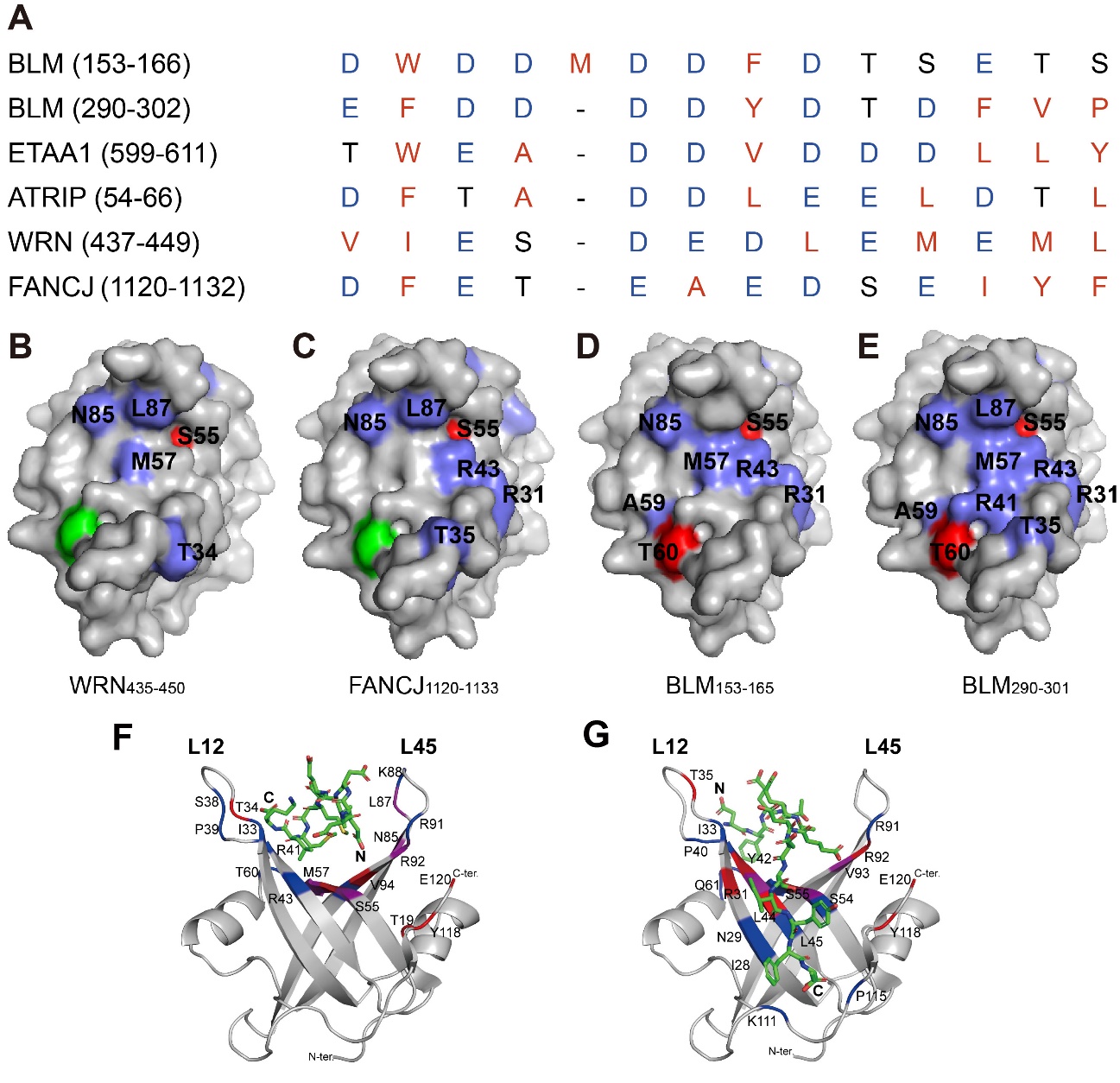


Figure S4. (A) Sequence alignment of human BLM_153-166_, BLM_290-302_, ETAA_599-611_, ATRIP_54-66_, WRN_437-449_, and FANCJ_1120-1132_. Hydrophobic residues are shown in red and acidic residues are shown in blue. Binding surfaces of RPA70N for (B) WRN_435-450_, (C) FANCJ_1120-1133_, (D) BLM_153-165_, and (E) BLM_290-301_ are shown in blue. S55 and T60 are colored in red. (B-C) T60, which is not perturbed in WRN_435-450_ and FANCJ_1120-1133_ binding, is shown in green. (F-G) In model 1 of each complex, residues perturbed by more than one standard deviation above the average in the NMR experiments and within 4.5 Å in the docking are colored in purple. Residues that only have Δδ_avg_ more than one standard deviation above the average are marked in red. Residues with only a distance of ≤ 4.5 Å in the docking are marked in blue.

**Supplementary methods**

Docking

Modeling of the RPA70N-WRN_441-450_ and RPA70N-FANCJ_1120-1133_ complexes was performed on the CABS-dock Web server^1^. We used the crystal structure of RPA70N (RCSB ID: 2B29) as the starting coordinates, and coordinates for the peptides were generated by the server based on each peptide sequence^2^. The detailed process was described previously^3^.

**Supplementary table**

Table S1. Details of Structural Clustering for RPA70N-WRN_441-450_ complex.

| Cluster name | Cluster density | Average cluster RMSD (Å) | Max RMSD | Peptide-RMSD from final model (Å) ^a^ | Number of elements |
| --- | --- | --- | --- | --- | --- |
| 1 | 36.57 | 2.73 | 6.98 | 2.71 ± 0.69 | 100 |
| 2 | 35.44 | 4.88 | 12.19 | 4.58 ± 1.10 | 173 |
| 3 | 19.00 | 4.37 | 11.48 | 6.81 ± 1.25 | 83 |
| 4 | 18.26 | 7.118 | 16.89 | 6.31 ± 1.78 | 130 |
| 5 | 14.78 | 5.752 | 23.72 | 12.68 ± 2.38 | 85 |
| 6 | 14.38 | 12.58 | 32.18 | 15.99 ± 4.36 | 181 |
| 7 | 10.02 | 7.188 | 18.25 | 12.35 ± 1.85 | 72 |
| 8 | 6.33 | 10.90 | 24.60 | 10.38 ± 2.54 | 69 |
| 9 | 3.91 | 14.58 | 33.45 | 11.96 ± 6.39 | 57 |
| 10 | 2.99 | 16.75 | 35.10 | 14.42 ± 3.90 | 50 |

^a^ Peptide-RMSD from the final model (RMSD calculated on the peptide after superimposition of receptor molecules (RPA70N, in our case)) for each cluster was analyzed using VMD v. 1.9.3^4^.

Table S2. Details of Structural Clustering for RPA70N-FANCJ_1120-1133_ complex.

| Cluster name | Cluster density | Average cluster RMSD (Å) | Max RMSD | Peptide-RMSD from final model (Å) ^a^ | Number of elements |
| --- | --- | --- | --- | --- | --- |
| 1 | 48.28 | 4.02 | 33.29 | 3.30 ± 3.41 | 194 |
| 2 | 28.00 | 4.71 | 17.38 | 10.02 ± 1.41 | 132 |
| 3 | 21.00 | 5.14 | 14.15 | 7.78 ± 1.04 | 108 |
| 4 | 19.19 | 4.27 | 9.94 | 4.39 ± 1.21 | 82 |
| 5 | 18.80 | 6.44 | 15.53 | 6.41 ± 1.07 | 121 |
| 6 | 17.73 | 6.99 | 17.42 | 13.48 ± 1.08 | 124 |
| 7 | 11.61 | 6.46 | 14.98 | 6.65 ± 1.88 | 75 |
| 8 | 11.42 | 6.04 | 12.44 | 5.44 ± 0.99 | 69 |
| 9 | 5.58 | 10.94 | 21.96 | 9.16 ± 2.74 | 61 |
| 10 | 3.54 | 9.60 | 30.58 | 23.35 ± 3.99 | 34 |

^a^ Peptide-RMSD from the final model (RMSD calculated on the peptide after superimposition of receptor molecules (RPA70N, in our case)) for each cluster was analyzed using VMD v. 1.9.3^4^.

**References**

1. Kurcinski M, Jamroz M, Blaszczyk M, Kolinski A & Kmiecik S. CABS-dock web server for the flexible docking of peptides to proteins without prior knowledge of the binding site. *Nucleic Acids Res.* **43**, W419–W424 (2015).

2. Bochkareva, E. *et al.* Single-stranded DNA mimicry in the p53 transactivation domain interaction with replication protein A. *Proc. Natl. Acad. Sci.* **102**, 15412–15417 (2005).

3. Kang, D. *et al.* Interaction of replication protein A with two acidic peptides from human Bloom syndrome protein. *FEBS Lett.* **592**, 547–558 (2018).

4. Humphrey W, Dalke A, & Schulten K VMD: visual molecular dynamics *J. Mol. Graph*. **14**, 27–38 (1996).
